# Bis(acetato-κ*O*)[*N*,*N*,*N*′,*N*′-tetra­methyl­ethane-1,2-diamine-κ^2^ *N*,*N*′]copper(II)

**DOI:** 10.1107/S1600536808002584

**Published:** 2008-01-30

**Authors:** J. Chris Slootweg, Peter Chen

**Affiliations:** a Laboratorium für Organische Chemie, Eidgenössische Technische Hochschule (ETH) Zürich, Wolfgang-Pauli-Strasse 10, 8093 Zürich, Switzerland

## Abstract

In the title compound, [Cu(C_2_H_3_O_2_)_2_(C_6_H_16_N_2_)], the Cu^II^ atom is coordinated by two N atoms from the chelating *N*,*N*,*N*′,*N*′-tetra­methyl­ethane-1,2-diamine ligand and two O atoms from two acetate anions in a distorted square-planar geometry. In addition, there are longer contacts between Cu and the second O atom of each acetate ligand, which could be considered to complete a distorted octa­hedral geometry. The mol­ecules in the crystal structure are connected *via* inter­molecular C—H⋯O hydrogen-bonding contacts.

## Related literature

For general background, see: Slootweg & Chen (2006 ▶); Gerdes & Chen (2004 ▶); Gerdes (2004 ▶). For related structures, see: Dalai *et al.* (2002 ▶); Margraf *et al.* (2005 ▶); Devereux *et al.* (2007 ▶); Brown *et al.* (2002 ▶).
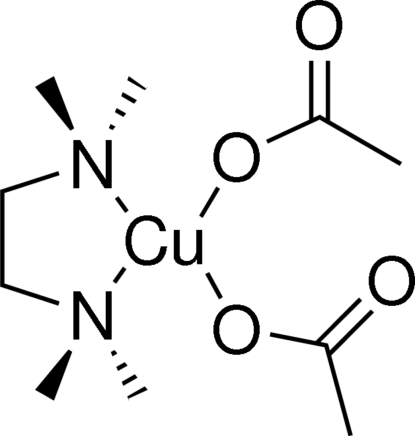


## Experimental

### 

#### Crystal data

[Cu(C_2_H_3_O_2_)_2_(C_6_H_16_N_2_)]
*M* *_r_* = 297.84
Monoclinic, 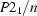

*a* = 8.0201 (5) Å
*b* = 15.9153 (10) Å
*c* = 10.8536 (7) Å
β = 90.910 (3)°
*V* = 1385.20 (15) Å^3^
*Z* = 4
Mo *K*α radiation
μ = 1.58 mm^−1^
*T* = 220 (2) K
0.21 × 0.19 × 0.15 mm

#### Data collection

Nonius KappaCCD area-detector diffractometer
Absorption correction: none
4704 measured reflections
2711 independent reflections
2321 reflections with *I* > 2σ(*I*)
*R*_int_ = 0.040

#### Refinement

*R*[*F* ^2^ > 2σ(*F* ^2^)] = 0.040
*wR*(*F* ^2^) = 0.106
*S* = 1.05
2711 reflections
183 parameters
H-atom parameters constrained
Δρ_max_ = 0.53 e Å^−3^
Δρ_min_ = −0.45 e Å^−3^

### 

Data collection: *COLLECT* (Nonius, 2000 ▶); cell refinement: *SCALEPACK* (Otwinowski & Minor, 1997 ▶); data reduction: *DENZO* (Otwinowski & Minor, 1997 ▶) and *SCALEPACK*; program(s) used to solve structure: *SIR97* (Altomare *et al.*, 1999 ▶); program(s) used to refine structure: *SHELXL97* (Sheldrick, 2008 ▶); molecular graphics: *PLATON* (Spek, 2003 ▶); software used to prepare material for publication: *SHELXL97*.

## Supplementary Material

Crystal structure: contains datablocks I, global. DOI: 10.1107/S1600536808002584/si2074sup1.cif

Structure factors: contains datablocks I. DOI: 10.1107/S1600536808002584/si2074Isup2.hkl

Additional supplementary materials:  crystallographic information; 3D view; checkCIF report

## Figures and Tables

**Table:** 

Cu1—O14	1.9797 (19)
Cu1—O10	1.9813 (19)
Cu1⋯O12	2.509 (2)
Cu1⋯O16	2.531 (2)
Cu1—N2	2.037 (2)
Cu1—N5	2.047 (2)

**Table:** 

O14—Cu1—O10	92.08 (9)
O14—Cu1—N2	164.30 (9)
O10—Cu1—N2	93.12 (9)
O14—Cu1—N5	92.40 (9)
O10—Cu1—N5	165.18 (9)
N2—Cu1—N5	86.30 (9)

**Table 2:** Hydrogen-bond geometry (Å, °)

*D*—H⋯*A*	*D*—H	H⋯*A*	*D*⋯*A*	*D*—H⋯*A*
C3—H3*A*⋯O12^i^	0.98	2.34	3.281 (4)	160
C4—H4*A*⋯O16^ii^	0.98	2.50	3.475 (4)	176
C13—H13*C*⋯O16^iii^	0.97	2.54	3.507 (4)	173
C17—H17*C*⋯O12^iv^	0.97	2.58	3.542 (4)	170

Symmetry codes: (i) 
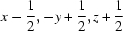
; (ii) 
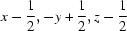
; (iii) 
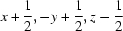
; (iv) 
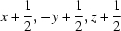
.

**Fig. 1.**
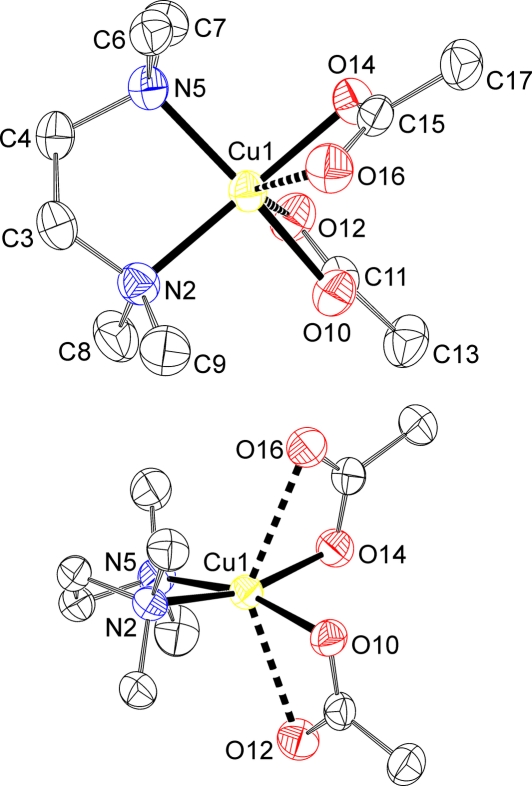
Displacement ellipsoid of [(TMEDA)Cu(OAc)2] with ellipsoids drawn at the 50% probability level. Weak interactions in the metal coordination sphere are shown as dotted lines. Hydrogen atoms are omitted for clarity.

## supplementary crystallographic information

### Comment

The aspiration of our work is to gain insight into the underlying mechanisms of the catalytic transformations of hydrocarbons by C—H bond activation (Slootweg & Chen, 2006; Gerdes & Chen, 2004) and subsequent oxidative coupling using heterobimetallic catalysis (Gerdes, 2004). Well defined platinum or palladium catalysts are suited for the C—H activation, whearas a copper-catalyzed coupling cycle is ideal for the C—*X* bond forming step. The intersection of the two cycles, that is transmetallation of the hydrocarbon group from platinum/palladium to copper, is poorly understood while being decisive for the outcome of the reaction. To connect two different catalyticallly active metal fragments, bridging acetate ligands are ideally suited (Gerdes, 2004) and therefore deserve our current attention. The title complex, [(TMEDA)Cu(OAc)_2_] (TMEDA = tetramethylethane-1,2-diamine), is a promising building block for the generation of the mixed [(TMEDA)Cu(κ^1^-acetate)(µ-acetate)*ML*_n_] complexes.

In the mononuclear title complex, the copper(II) atom is in a distorted, square-planar coordination geometry (Fig. 1, Table 1) and bonded to the bidentate tetramethylethane-1,2-diamine ligand [Cu—N 2.037 (2), 2.047 (2) Å] and to the two acetate anions (Dalai *et al.*, 2002; Margraf *et al.*, 2005). The acetato groups in [(TMEDA)Cu(OAc)_2_] are mono-coordinating [Cu—O 1.979 (2), 1.9810 (19) Å] (Devereux *et al.*, 2007), but the second oxygen atom of each ligand shows an additional weak interaction with the copper atom [Cu—O 2.509 (2), 2.531 (2) Å], and could be considered to complete a distorted octahedral geometry (Dalai *et al.*, 2002). A similar situation is observed for the zinc analogue, [(TMEDA)Zn(OAc)_2_], which displays more pronounced κ^2^-coordination of the acetates [Zn—O 2.052 (2), 2.353 (4) Å] (Brown *et al.*, 2002).

The molecules in the crystal are connected *via* hydrogen bonding. There are four short intermolecular C—H···O contacts with O···H distances between 2.34 and 2.58 Å and C—H···O angles between 160 and 176° (Table 2).

### Experimental

General Procedures. ESI-MS measurements were performed on a Finnigan MAT TSQ Quantum triple-quad mass spectrometer equipped with electrospray sources. Elemental analyses were performed by the Microanalytical Laboratory of the Laboratorium für Organische Chemie, ETH Zürich.

The title compound was obtained as follows. To a suspension of copper(II)acetate (1.78 g, 9.78 mmol) in MeOH (40 ml) an equimolar amount of TMEDA (1.48 ml, 9.78 mmol) was added and the reaction mixture was stirred for 18 h at room temperature yielding a deep blue solution. After filtration, the solvent was removed *in vacuo* yielding analytically pure [(TMEDA)Cu(OAc)_2_] (2.64 g, 91%) as a blue powder. Crystallization from THF at room temperature yielded blue plates suitable for X-ray crystallography. *M*.p. 178 °C (decomp.). MS (ESI, positive ions, DCM) m/*z* (%): 238 (100) [*M* - O_2_CCH_3_]^+^. Elem. anal.: Found C, 40.18; H, 7.15; N, 9.41. Calcd. for C_10_H_22_N_2_O_4_Cu: C, 40.33; H, 7.44; N, 9.41.

### Refinement

The structure was refined by full-matrix least-squares analysis using an isotropic extinction correction. All non H-atoms were refined anisotropically, H-atoms isotropically, whereby H-positions are based on stereochemical considerations. For CH_3_ groups, C—H distances are 0.97 Å and *U*_iso_(H) = 1.5U(eq) on the respective C-atom, while for CH_2_ groups, the corresponding values are 0.98 Å and 1.2U(eq), respectively.

### Crystal data

**Table:**

[Cu(C_2_H_3_O_2_)_2_(C_6_H_16_N_2_)]	*F*_000_ = 628
*M**_r_* = 297.84	*D*_x_ = 1.428 Mg m^−^^3^
Monoclinic, *P*2_1_/*n*	Melting point: 178 K
Hall symbol: -P 2yn	Mo *K*α radiation λ = 0.7107 Å
*a* = 8.0201 (5) Å	Cell parameters from 10930 reflections
*b* = 15.9153 (10) Å	θ = 3.2–26.0º
*c* = 10.8536 (7) Å	µ = 1.58 mm^−^^1^
β = 90.910 (3)º	*T* = 220 (2) K
*V* = 1385.20 (15) Å^3^	Cut fragment, blue
*Z* = 4	0.21 × 0.19 × 0.15 mm

### Data collection

**Table:**

Nonius KappaCCD area-detector diffractometer	2321 reflections with *I* > 2σ(*I*)
Radiation source: fine-focus sealed tube	*R*_int_ = 0.040
Monochromator: graphite	θ_max_ = 26.0º
*T* = 220(2) K	θ_min_ = 3.2º
φ and ω scans with κ offsets	*h* = −9→9
Absorption correction: none	*k* = −16→19
4704 measured reflections	*l* = −13→13
2711 independent reflections	

### Refinement

**Table:**

Refinement on *F*^2^	Hydrogen site location: inferred from neighbouring sites
Least-squares matrix: full	H-atom parameters constrained
*R*[*F*^2^ > 2σ(*F*^2^)] = 0.040	*w* = 1/[σ^2^(*F*_o_^2^) + (0.0446*P*)^2^ + 0.7399*P*] where *P* = (*F*_o_^2^ + 2*F*_c_^2^)/3
*wR*(*F*^2^) = 0.106	(Δ/σ)_max_ = 0.006
*S* = 1.06	Δρ_max_ = 0.53 e Å^−^^3^
2711 reflections	Δρ_min_ = −0.45 e Å^−^^3^
183 parameters	Extinction correction: SHELXL97 (Sheldrick, 2008), Fc^*^=kFc[1+0.001xFc^2^λ^3^/sin(2θ)]^-1/4^
Primary atom site location: structure-invariant direct methods	Extinction coefficient: 0.0108 (18)
Secondary atom site location: difference Fourier map	

### Special details

**Table:**

Geometry. All e.s.d.'s (except the e.s.d. in the dihedral angle between two l.s. planes) are estimated using the full covariance matrix. The cell e.s.d.'s are taken into account individually in the estimation of e.s.d.'s in distances, angles and torsion angles; correlations between e.s.d.'s in cell parameters are only used when they are defined by crystal symmetry. An approximate (isotropic) treatment of cell e.s.d.'s is used for estimating e.s.d.'s involving l.s. planes.
Refinement. Refinement of *F*^2^ against ALL reflections. The weighted *R*-factor *wR* and goodness of fit *S* are based on *F*^2^, conventional *R*-factors *R* are based on *F*, with *F* set to zero for negative *F*^2^. The threshold expression of *F*^2^ > σ(*F*^2^) is used only for calculating *R*-factors(gt) *etc*. and is not relevant to the choice of reflections for refinement. *R*-factors based on *F*^2^ are statistically about twice as large as those based on *F*, and *R*- factors based on ALL data will be even larger.

### Fractional atomic coordinates and isotropic or equivalent isotropic displacement parameters (Å^2^)

**Table:**

	*x*	*y*	*z*	*U*_iso_*/*U*_eq_	
Cu1	0.53786 (4)	0.26068 (2)	0.13564 (3)	0.03199 (13)	
N2	0.3491 (3)	0.18731 (15)	0.1983 (2)	0.0345 (5)	
C3	0.1951 (4)	0.23935 (18)	0.1898 (3)	0.0382 (7)	
H3A	0.1903	0.2777	0.2603	0.058 (11)*	
H3B	0.0964	0.2031	0.1909	0.051 (9)*	
C4	0.1978 (4)	0.28884 (19)	0.0717 (3)	0.0389 (7)	
H4A	0.1912	0.2506	0.0010	0.038 (9)*	
H4B	0.1016	0.3268	0.0675	0.055 (11)*	
N5	0.3551 (3)	0.33834 (15)	0.0673 (2)	0.0374 (5)	
C6	0.3407 (4)	0.4158 (2)	0.1401 (4)	0.0529 (9)	
H6A	0.2490	0.4494	0.1078	0.079 (12)*	
H6B	0.4436	0.4476	0.1351	0.044 (9)*	
H6C	0.3201	0.4016	0.2254	0.066 (12)*	
C7	0.3938 (4)	0.3596 (3)	−0.0616 (3)	0.0562 (9)	
H7A	0.3075	0.3960	−0.0952	0.079 (14)*	
H7B	0.3988	0.3085	−0.1101	0.073 (12)*	
H7C	0.5005	0.3881	−0.0642	0.059 (11)*	
C8	0.3279 (4)	0.10915 (18)	0.1261 (3)	0.0414 (7)	
H8A	0.2369	0.0766	0.1595	0.062 (10)*	
H8B	0.4299	0.0765	0.1308	0.048 (9)*	
H8C	0.3029	0.1231	0.0408	0.070 (13)*	
C9	0.3865 (4)	0.1654 (2)	0.3283 (3)	0.0448 (7)	
H9A	0.3981	0.2164	0.3765	0.053 (10)*	
H9B	0.4896	0.1336	0.3330	0.058 (10)*	
H9C	0.2963	0.1317	0.3605	0.081 (13)*	
O10	0.7070 (2)	0.17122 (13)	0.15991 (18)	0.0385 (5)	
C11	0.7292 (3)	0.14698 (17)	0.0489 (3)	0.0349 (6)	
O12	0.6444 (3)	0.17379 (14)	−0.03924 (19)	0.0465 (5)	
C13	0.8650 (4)	0.0828 (2)	0.0276 (3)	0.0492 (8)	
H13A	0.8172	0.0335	−0.0118	0.083 (13)*	
H13B	0.9156	0.0669	0.1060	0.104 (18)*	
H13C	0.9493	0.1068	−0.0250	0.096 (15)*	
O14	0.7116 (2)	0.34802 (13)	0.11797 (18)	0.0405 (5)	
C15	0.7388 (4)	0.36997 (18)	0.2298 (3)	0.0369 (6)	
O16	0.6567 (3)	0.34218 (14)	0.31695 (19)	0.0449 (5)	
C17	0.8749 (4)	0.4340 (2)	0.2530 (4)	0.0503 (8)	
H17A	0.8266	0.4850	0.2858	0.078 (12)*	
H17B	0.9300	0.4466	0.1762	0.084 (16)*	
H17C	0.9557	0.4115	0.3117	0.091 (13)*	

### Atomic displacement parameters (Å^2^)

**Table:**

	*U*^11^	*U*^22^	*U*^33^	*U*^12^	*U*^13^	*U*^23^
Cu1	0.0299 (2)	0.0361 (2)	0.0299 (2)	0.00122 (13)	−0.00067 (14)	−0.00086 (13)
N2	0.0332 (12)	0.0405 (12)	0.0295 (12)	0.0007 (10)	−0.0031 (9)	−0.0032 (10)
C3	0.0315 (14)	0.0484 (16)	0.0346 (15)	0.0014 (12)	0.0018 (12)	−0.0055 (12)
C4	0.0351 (15)	0.0467 (16)	0.0348 (15)	0.0062 (13)	−0.0063 (12)	−0.0057 (13)
N5	0.0379 (13)	0.0390 (12)	0.0352 (13)	0.0029 (10)	−0.0005 (10)	0.0015 (10)
C6	0.0446 (18)	0.0394 (16)	0.074 (3)	0.0082 (14)	−0.0051 (17)	−0.0102 (16)
C7	0.054 (2)	0.071 (2)	0.0435 (19)	0.0032 (18)	−0.0009 (16)	0.0163 (18)
C8	0.0379 (15)	0.0382 (15)	0.0481 (19)	−0.0035 (12)	−0.0016 (13)	−0.0061 (13)
C9	0.0479 (18)	0.0558 (18)	0.0306 (15)	0.0010 (15)	−0.0045 (13)	0.0079 (14)
O10	0.0361 (11)	0.0447 (11)	0.0347 (11)	0.0060 (9)	−0.0011 (8)	−0.0023 (9)
C11	0.0273 (13)	0.0368 (14)	0.0405 (16)	−0.0042 (11)	0.0034 (11)	0.0005 (12)
O12	0.0471 (12)	0.0582 (13)	0.0341 (11)	0.0019 (10)	−0.0039 (9)	0.0026 (10)
C13	0.0395 (17)	0.0447 (17)	0.064 (2)	0.0024 (13)	0.0077 (16)	−0.0079 (16)
O14	0.0384 (11)	0.0465 (11)	0.0366 (11)	−0.0045 (9)	0.0025 (9)	−0.0017 (9)
C15	0.0315 (14)	0.0392 (15)	0.0399 (16)	0.0031 (11)	−0.0014 (12)	−0.0018 (12)
O16	0.0441 (12)	0.0536 (12)	0.0371 (11)	−0.0046 (10)	0.0056 (9)	−0.0008 (10)
C17	0.0405 (17)	0.0480 (17)	0.062 (2)	−0.0092 (14)	0.0007 (16)	−0.0118 (16)

### Geometric parameters (Å, °)

**Table:**

Cu1—O14	1.9797 (19)	C4—H4A	0.98
Cu1—O10	1.9813 (19)	C4—H4B	0.98
Cu1—O12	2.509 (2)	C6—H6A	0.97
Cu1—O16	2.531 (2)	C6—H6B	0.97
Cu1—N2	2.037 (2)	C6—H6C	0.97
Cu1—N5	2.047 (2)	C7—H7A	0.97
N2—C8	1.478 (4)	C7—H7B	0.97
N2—C9	1.480 (4)	C7—H7C	0.97
N2—C3	1.489 (4)	C8—H8A	0.97
C3—C4	1.505 (4)	C8—H8B	0.97
C4—N5	1.489 (4)	C8—H8C	0.97
N5—C6	1.470 (4)	C9—H9A	0.97
N5—C7	1.477 (4)	C9—H9B	0.97
O10—C11	1.280 (4)	C9—H9C	0.97
C11—O12	1.241 (3)	C13—H13A	0.97
C11—C13	1.514 (4)	C13—H13B	0.97
O14—C15	1.278 (4)	C13—H13C	0.97
C15—O16	1.243 (4)	C17—H17A	0.97
C15—C17	1.512 (4)	C17—H17B	0.97
C3—H3A	0.98	C17—H17C	0.97
C3—H3B	0.98		
			
O14—Cu1—O10	92.08 (9)	H4A—C4—H4B	108
O14—Cu1—N2	164.30 (9)	N5—C6—H6A	109
O10—Cu1—N2	93.12 (9)	N5—C6—H6B	109
O14—Cu1—N5	92.40 (9)	N5—C6—H6C	109
O10—Cu1—N5	165.18 (9)	H6A—C6—H6B	109
N2—Cu1—N5	86.30 (9)	H6A—C6—H6C	109
C8—N2—C9	109.1 (2)	H6B—C6—H6C	109
C8—N2—C3	110.3 (2)	N5—C7—H7A	109
C9—N2—C3	110.2 (2)	N5—C7—H7B	109
C8—N2—Cu1	112.67 (18)	N5—C7—H7C	109
C9—N2—Cu1	108.25 (18)	H7A—C7—H7B	109
C3—N2—Cu1	106.29 (17)	H7A—C7—H7C	110
N2—C3—C4	108.7 (2)	H7B—C7—H7C	109
N5—C4—C3	109.1 (2)	N2—C8—H8A	109
C6—N5—C7	109.7 (3)	N2—C8—H8B	109
C6—N5—C4	110.7 (2)	N2—C8—H8C	109
C7—N5—C4	110.0 (2)	H8A—C8—H8B	109
C6—N5—Cu1	111.94 (19)	H8A—C8—H8C	109
C7—N5—Cu1	108.74 (19)	H8B—C8—H8C	110
C4—N5—Cu1	105.74 (17)	N2—C9—H9A	109
C11—O10—Cu1	101.36 (17)	N2—C9—H9B	109
O12—C11—O10	122.6 (3)	N2—C9—H9C	109
O12—C11—C13	120.1 (3)	H9A—C9—H9B	110
O10—C11—C13	117.3 (3)	H9A—C9—H9C	110
C15—O14—Cu1	102.12 (18)	H9B—C9—H9C	109
O16—C15—O14	122.7 (3)	C11—C13—H13A	109
O16—C15—C17	120.2 (3)	C11—C13—H13B	110
O14—C15—C17	117.0 (3)	C11—C13—H13C	110
N2—C3—H3A	110	H13A—C13—H13B	109
N2—C3—H3B	110	H13A—C13—H13C	109
C4—C3—H3A	110	H13B—C13—H13C	109
C4—C3—H3B	110	C15—C17—H17A	110
H3A—C3—H3B	108	C15—C17—H17B	109
N5—C4—H4A	110	C15—C17—H17C	109
N5—C4—H4B	110	H17A—C17—H17B	109
C3—C4—H4A	110	H17A—C17—H17C	110
C3—C4—H4B	110	H17B—C17—H17C	109
			
O14—Cu1—N2—C8	−167.6 (3)	N2—Cu1—N5—C6	106.3 (2)
O10—Cu1—N2—C8	−58.42 (19)	O14—Cu1—N5—C7	63.3 (2)
N5—Cu1—N2—C8	106.75 (19)	O10—Cu1—N5—C7	−44.1 (5)
O14—Cu1—N2—C9	−46.9 (4)	N2—Cu1—N5—C7	−132.3 (2)
O10—Cu1—N2—C9	62.3 (2)	O14—Cu1—N5—C4	−178.58 (17)
N5—Cu1—N2—C9	−132.6 (2)	O10—Cu1—N5—C4	74.0 (4)
O14—Cu1—N2—C3	71.5 (4)	N2—Cu1—N5—C4	−14.25 (17)
O10—Cu1—N2—C3	−179.34 (17)	O14—Cu1—O10—C11	−90.25 (17)
N5—Cu1—N2—C3	−14.18 (18)	N2—Cu1—O10—C11	104.56 (18)
C8—N2—C3—C4	−82.3 (3)	N5—Cu1—O10—C11	17.2 (4)
C9—N2—C3—C4	157.2 (2)	Cu1—O10—C11—O12	−5.8 (3)
Cu1—N2—C3—C4	40.1 (3)	Cu1—O10—C11—C13	174.5 (2)
N2—C3—C4—N5	−55.2 (3)	O10—Cu1—O14—C15	−87.43 (18)
C3—C4—N5—C6	−81.1 (3)	N2—Cu1—O14—C15	21.9 (4)
C3—C4—N5—C7	157.5 (3)	N5—Cu1—O14—C15	106.71 (19)
C3—C4—N5—Cu1	40.3 (3)	Cu1—O14—C15—O16	−4.8 (3)
O14—Cu1—N5—C6	−58.0 (2)	Cu1—O14—C15—C17	176.6 (2)
O10—Cu1—N5—C6	−165.4 (3)		

### Hydrogen-bond geometry (Å, °)

**Table:**

*D*—H···*A*	*D*—H	H···*A*	*D*···*A*	*D*—H···*A*
C3—H3A···O12^i^	0.98	2.34	3.281 (4)	160
C4—H4A···O16^ii^	0.98	2.50	3.475 (4)	176
C13—H13C···O16^iii^	0.97	2.54	3.507 (4)	173
C17—H17C···O12^iv^	0.97	2.58	3.542 (4)	170

Symmetry codes: (i) *x*−1/2, −*y*+1/2, *z*+1/2; (ii) *x*−1/2, −*y*+1/2, *z*−1/2; (iii) *x*+1/2, −*y*+1/2, *z*−1/2; (iv) *x*+1/2, −*y*+1/2, *z*+1/2.
